# Survival in pulmonary fibrosis combined with emphysema: likely defined by characteristics of specific patient subpopulations

**DOI:** 10.1186/1755-1536-4-17

**Published:** 2011-07-25

**Authors:** Nevins W Todd, Sergei P Atamas

**Affiliations:** 1 University of Maryland School of Medicine and Baltimore VA Medical Center, Baltimore, MD, USA

## Abstract

Authors' Reply to letter from Cottin et al.

Please see related letter http://www.fibrogenesis.com/content/4/1/16

## To the Editor

We thank Cottin *et al*. for their interest in our work [1], and for sharing their engaging thoughts. They noted that our results appear to be in contrast with previous studies, and cite three references in support [2-4]. We agree that on the surface, there may be apparent disagreement with some previous results, but in-depth analysis does not suggest inconsistency between findings made by various groups of investigators.

In the study by Cottin *et al*. [2], patients with combined pulmonary fibrosis and emphysema had a median survival of 6.1 years, a very similar survival time to our patients with advanced centrilobular emphysema combined with pulmonary fibrosis, who had a median survival of 6.2 years (75 months). Thus, these two reports are in full agreement on the duration of patient survival. However, contrasting the findings of both of these studies is another study of patients with idiopathic pulmonary fibrosis (IPF) and emphysema [3]. Of important note in this study, patients in the IPF and emphysema group had a median history of smoking of 5 pack-years, whereas in our study, patients with advanced emphysema and pulmonary fibrosis had a median 40 pack-year history of smoking. Because heavy cigarette smoking has been suggested to play a causative role in patients who develop emphysema combined with pulmonary fibrosis, it is not surprising that the outcomes differed between this study and our report. Further input on survival in patients with pulmonary fibrosis and emphysema comes from Antoniou *et al*. [4], who reported that 'findings were unchanged when the presence of emphysema ... was taken into account in survival analysis, with the presence of emphysema having no influence on survival.' However, this study focused not on patients with emphysema combined with fibrosis, but rather on comparing patients with IPF who were smokers versus those who were non-smokers. Smoking and emphysema are certainly associated, but these are two separate populations of patients that should not be equated.

Taking together all the observations in these four studies [1-4], it appears that generalized conclusions on survival of patients with emphysema combined with pulmonary fibrosis should be avoided, as survival varies depending on the specific characteristics of patient subpopulations being studied. In our work specifically [1], the mere presence of emphysema in patients with pulmonary fibrosis did not equate to better survival, and only a subpopulation of our patients (those with an advanced centrilobular component to their emphysema) survived longer, as we reported in detail.

The presence of non-specific interstitial pneumonia (NSIP) in our group of patients with advanced emphysema and pulmonary fibrosis was discussed, although the patterns of usual interstitial pneumonia (UIP)/NSIP were not significantly different between the groups in our study. The attempt to extrapolate percentages of pathologic patterns to patients from whom a surgical lung biopsy was not obtained is inconsistent with the scientific method. A strength of our study is the large portion of patients (70%) from whom surgical lung tissue was available for pathologic examination, both in the entire cohort and in the advanced emphysema with fibrosis group. This is in contrast to two previous studies of patients with combined pulmonary fibrosis and emphysema, in which only approximately 15% of patients had histological analysis of surgical or explanted lung tissue [2,5].

The handling, with regard to survival analysis, of patients in our study who underwent lung transplantation is also discussed. As suggested, we censored patients who underwent lung transplantation, and performed survival analyses. Comparison of survival curves by log rank tests showed improved survival in patients with pulmonary fibrosis and advanced emphysema compared with patients with pulmonary fibrosis without emphysema (*P *= 0.014) and patients with pulmonary fibrosis and trivial emphysema (*P *= 0.015). There was no survival difference between patients with pulmonary fibrosis without emphysema and patients with fibrosis and trivial emphysema (*P *= 0.776). Therefore, our original findings remained the same when patients who underwent transplantation were censored for the statistical analysis.

Cottin *et al*. question the severity of fibrosis in our advanced emphysema and fibrosis group, based on an interpretation of our DL_CO _(diffusing capacity of the lung for carbon monoxide) findings. It is possible, in the absence of data on total lung collagen, that the lack of difference in DL_CO _between groups could be due to differences in the amount of fibrosis. Direct estimation of total lung collagen in humans is obviously challenging, and we thus used an accepted visual fibrosis score, as described [1]. It is, however, important to keep in mind that although both emphysema and fibrosis cause reductions in DL_CO_, there is often a loose statistical association between severity of emphysema or fibrosis determined by visual scores and the severity of the reductions in DL_CO _[6,7]. The expectation of a strict correlation between emphysema or fibrosis scores and DL_CO _is not justified based on these observations.

Overall, our study does not appear to be inconsistent with the existing body of literature concerning patients with emphysema combined with pulmonary fibrosis, and it expands further the factual knowledge in this field. There are certainly aspects of our study that are open to scientific speculation, but we identified those (for example, a possibility that pulmonary inflammation resulting from the presence of emphysema may be partially protective against the deleterious effects of pulmonary fibrosis) in the discussion section of our report.

In summary, emphysema combined with pulmonary fibrosis is likely to be a heterogenous entity, in which survival is defined by characteristics of specific patient subpopulations. In our cohort of patients, survival was improved when advanced centrilobular emphysema was present in patients with pulmonary fibrosis.
